# Quality of reporting of complex healthcare interventions and applicability of the CReDECI list - a survey of publications indexed in PubMed

**DOI:** 10.1186/1471-2288-13-125

**Published:** 2013-10-19

**Authors:** Ralph Möhler, Gabriele Bartoszek, Gabriele Meyer

**Affiliations:** 1 School of Nursing Science, Faculty of Health, Witten/Herdecke University, Stockumer Strasse 12, 58453, Witten, Germany; 2 Institute of Health and Nursing Science, Martin-Luther-University Halle-Wittenberg, Magdeburger Strasse 8, 06112, Halle/Saale, Germany

**Keywords:** Complex interventions, Evidence-based nursing, Reporting guideline, Research design, Review

## Abstract

**Background:**

The development and evaluation of complex interventions in healthcare has obtained increased awareness. The Medical Research Council’s (MRC) framework for the development and evaluation of complex interventions and its update offers guidance for researchers covering the phases development, feasibility/piloting, and evaluation. Comprehensive reporting of complex interventions enhances transparency and is essential for researchers and policy-makers. Recently, a set of 16 criteria for reporting complex interventions in healthcare (CReDECI) was published. The aim of this study is to evaluate the reporting quality in publications of complex interventions adhering to either the first or the updated MRC framework, and to evaluate the applicability of CReDECI.

**Methods:**

A systematic PubMed search was conducted. Two reviewers independently checked titles and abstracts for inclusion. Trials on complex interventions adhering to the MRC framework and including an evaluation study in English and German were included. For all included trials and for all publications which reported on phases prior to the evaluation study, related publications were identified via forward citation tracking. The quality of reporting was assessed independently by two reviewers using CReDECI. Inter-rater agreement and time needed to complete the assessment were determined.

**Results:**

Twenty-six publications on eight trials were included. The number of publications per trial ranged from 1 to 6 (mean 3.25). The trials demonstrate a good reporting quality for the criteria referring to the development and feasibility/piloting. For the criteria addressing the introduction of the intervention and the evaluation, quality of reporting varied widely. Two trials fulfilled 7 and 8 items respectively, five trials fulfilled one to five items and one trial offered no information on any item. The mean number of items with differing ratings per trial was two. The time needed to rate a trial ranged from 30 to 90 minutes, depending on the number of publications.

**Conclusions:**

Adherence to the MRC framework seems to have a positive impact on the reporting quality on the development and piloting of complex interventions. Reporting on the evaluation could be improved. CReDECI is a practical instrument to check the reporting quality of complex interventions and could be used alongside design-specific reporting guidelines.

## Background

Researching complex interventions in healthcare and nursing has gained increased awareness following the publication of the UK Medical Research Council’s (MRC) framework for development and evaluation of complex interventions in 2000 [1] and its update in 2008 [2,3]. The MRC framework defined complex interventions as interventions comprising several components which may act either independently or inter-dependently [2]. However, there is no distinct boundary in the classification of whether an intervention is complex or not. Characteristics of complex interventions are, for example, the number of different professions or organisational levels targeted by the intervention and/or the degree of flexibility permitted for the intervention [4].

While the first framework [1] implied a linear approach comprising one preclinical and four clinical phases, the updated framework [2,3] builds on a circular model which gives a better reflection of the flexibility or even non-linearity of the research process. However, both versions cover the same methodological steps of development, piloting and evaluation of a complex intervention, and the long-term implementation after the complex intervention has demonstrated its effectiveness. Also, both frameworks emphasise that the development of a complex intervention should include a theoretical basis and a clear description of the intended change processes. Prior to the evaluation, the intervention should be piloted in the target setting and its feasibility has to be tested. The evaluation study should not only focus on effectiveness and cost-effectiveness but also include the evaluation of the intended change process by investigating the dose delivered, fidelity and reach of the intervention (process evaluation) [5-7]. Thus, the development and evaluation of complex interventions requires several studies using different methods and study designs [1-4].

Comprehensive reporting of all steps of the development and evaluation of a complex intervention is crucial. Sufficient information must be available for the judgement of the intervention’s clinical benefits, for replication, or for adaption of an intervention to different settings or countries [8-10]. However, several analyses revealed shortcomings of the reporting of core aspects of complex intervention research, e.g. regarding the theoretical basis and assumptions guiding the development and piloting of the intervention, and the description of intervention delivery [10-13]. These studies evaluated interventions on defined topics, e.g. care of people after stroke [13] and reduction of physical restraints in geriatric care [12], or specific methodological aspects of research on complex interventions [10,11]. An evaluation of the reporting quality of a sample of systematically identified complex interventions explicitly adhering to the MRC framework has not been performed so far.

We have recently published a list of specific criteria for reporting complex interventions in order to offer a structured guidance for researchers and authors covering the first three phases of the MRC framework [14]. In contrast to other reporting guidelines (e.g. CONSORT), CReDECI does not comprise design-specific items. The development and evaluation of complex interventions requires the use of different methodological approaches and the criteria list includes only items covering these specific methodological aspects [1-4,14]. Therefore, CReDECI should be used alongside established study design-specific reporting statements.

Employing the CReDECI criteria, the aim of the present study was to evaluate the reporting quality of the development and evaluation of complex interventions adhering to the MRC framework. The second aim was to test the applicability of the criteria list.

## Methods

### Literature search and study selection

All trials which adhered to the MRC framework were eligible for inclusion. We use the term ‘trial’ for the entire research process covering development, piloting and evaluation of a complex intervention and all related publications or reports. Adherence to the MRC framework was defined as citing either the first version [1] or the updated framework [3] as methodological guide for the development and evaluation of a complex intervention. According to the aim of our study to test the applicability of all CReDECI criteria, the included trials should have undergone a controlled evaluation study.

The following inclusion criteria were defined: The intervention was (I) labelled as ‘complex intervention’, (II) developed and evaluated adhering to the MRC framework, and (III) a controlled evaluation study was published. All citations retrieved by database searching and ‘snowballing’ techniques were screened independently by two reviewers (RM and GB) and checked for inclusion. We conducted a database search and used snowballing techniques to identify relevant publications since we expected several publications reporting on different phases of the same trial [15].

A systematic search in Medline (via PubMed) was performed in April 2012. Medline was searched because this database is the primary resource for clinicians and researchers and most journals publishing evaluation studies of health care interventions are covered. The search was limited to German and English publications of the last ten years. We explored several search strategies to select the adequate search terms. Finally, we used a combination of terms related to complex interventions and to the MRC framework (the complete search strategy is described in Additional file 1). Since we used additional snowballing technique to identify further relevant publications, we believe the search strategy employed to be sufficiently specific. In a second step, we conducted forward citation tracking (via Scopus and Google Scholar). For all citations identified by the database search which reported on the development and/or piloting of a complex intervention we checked whether a publication of an evaluation study was available. In a third step, we identified the associated publications of the included trials, e.g. publications on the interventions’ development.

### CReDECI

The CReDECI list [14] was developed based on a systematic literature review of methodological publications on complex intervention research and the updated MRC framework and reviewed by experts in the field. The criteria list is divided into three sections: development (n = 6 items), feasibility and piloting (n = 2 items), and introduction of the intervention and evaluation (n = 8 items). In contrast to most of the available reporting guidelines, the CReDECI list does not comprise items targeting a specific study-design, since the development and evaluation of complex interventions requires different study designs conducted subsequently or in parallel [2]. Instead, CReDECI focuses on criteria covering the core elements that are specific for the research of complex interventions. An overview of the criteria and their explanation is presented in Table 1.

**Table 1:** Overview of CReDECI

**No**	**Item**	**Explanation**
**First phase – Development**		
1	Description of the intervention’s underlying theoretical considerations	The theoretical basis of the intervention should be clearly stated. This includes the theory on which the intervention is founded as well as, if available, empirical evidence from studies in different settings or countries.
2	Description of all components of the intervention	Complex interventions contain several interacting components, which make up the intervention. All components should be clearly specified. A graphical presentation of the components might be useful.
3	Rationale for the selection of the intervention’s components	A description of the rationale for choosing the selected components should be given. If formerly successfully proven components have been excluded from the intervention, the rationale should be stated.
4	Illustration of any intended interactions between different components	In some cases different components are designed to support or to enhance the effect of other components. All expected reciprocal effects should be explained.
5	Rationale for the aim/essential functions of the intervention’s components, including the evidence whether the components are appropriate for achieving this goal	It is necessary to describe the aim or essential function of the intervention’s components rather than the content in detail, e.g. the content of an education programme may differ more between various countries than the aim or essential function.
6	Consideration of contextual factors and determinants of the setting in the modelling of the intervention	The intervention should be tailored to the target setting. This includes legal or political issues of a country as well as local conditions of the participating centres.
**Second phase – Feasibility and piloting**		
7	Information on pilot-testing	The intervention should have been pilot-tested in order to determine feasibility, acceptability, and practicability of the complex intervention. The pilot test should take into account the key uncertainties which have been identified during the development process.
8	In case of pilot-test: presentation of all relevant results and their impact on the modelling of the final intervention	Results of the pilot test and any subsequent modification of the intervention are highly relevant for other researchers in the field and should be published.
**Third phase – Introduction of the intervention and evaluation**		
9	Description of the control intervention (comparator)	Information on the characteristics of the control intervention (e.g. usual care or optimised usual care) should be given. It should be stated whether components of the intervention were accessible for the control group, whether a specific control intervention was delivered, or whether the control group did not receive any intervention. If the study took place in different centres, differences in usual care across centres should also be described.
10	If the study was conducted in different clusters or centres: description of a standardised implementation strategy throughout the centres	The implementation strategy should include methods to deal with the local conditions, e.g. education of persons in charge for the implementation, and methods to adjust the implementation process in order to maintain a standardised implementation of the intervention.
11	Description of all materials or tools used for the implementation of the intervention to allow a replication of the study	Complex interventions often comprise materials, e.g. brochures, checklists or flyers. These materials might influence the interventions’ effects and should be publicly accessible. In addition, the use of incentives may influence study adherence and should therefore be stated.
12	Description of an evaluation of the implementation process	Process evaluation is a prerequisite for determining the success of the intervention’s implementation and should be an integral part of the intervention’s evaluation.
13	Description of any deviation from the study protocol during the implementation process	In order to replicate the study, information on the actual delivery of the intervention and on any deviation from the study protocol concerning the implementation should be reported. Deviations and necessary adjustments of single components or the whole intervention during the implementation process should be published. Adjustments may have been necessary for a single centre, several clusters or the whole intervention group.
14	Description of facilitators or barriers revealed by the process evaluation which have influenced the interventions’ implementation	Any facilitators or barriers identified in the context of the process evaluation should be described. Information on facilitators or barriers may be derived both from staff and from the research team. Any interpretation of facilitators or barriers, e.g. in the discussion section of the publication, should be clearly separated in (1) information collected during the process evaluation and (2) information derived from other studies.
15	Description of unexpected interactions between components of the intervention and the environment in which the intervention was implemented	It should be stated if any change had been observed which may have been caused by the implementation of the intervention.
16	Description of costs or required resources for the intervention’s implementation	Information on costs or required resources necessary to implement the intervention should be available in the publication or as reference to an economic evaluation. Resources should include all expenses necessary for the intervention’s implementation, e.g. personnel costs, material or equipment.

### Data extraction

Two independent reviewers (RM, GB) applied CReDECI to all publications related to the respective included trials. For each item it was recorded whether sufficient information was provided in any publication. In order to distinguish between sufficient and insufficient information, the raters assessed whether the information provided was judged as comprehensive. The information to the individual items may have been included in several publications. However, if a minimum of comprehensive information was offered, the items were rated as fulfilled. We did not assess the degree of comprehensibility.

The results of the independent ratings were compared and agreement between both raters was checked. Thereafter, differences in the ratings were discussed in order to reach consensus. If results on planned parts of the trial were missing (e.g. economic evaluation or process evaluation), the corresponding author was contacted. The number of trials fulfilling the criteria was calculated. The absolute number of different ratings per trial was determined with regard to inter-rater reliability as well as to the time spent on completing the list (i.e. mean time of both raters).

## Results

**Figure 1:**
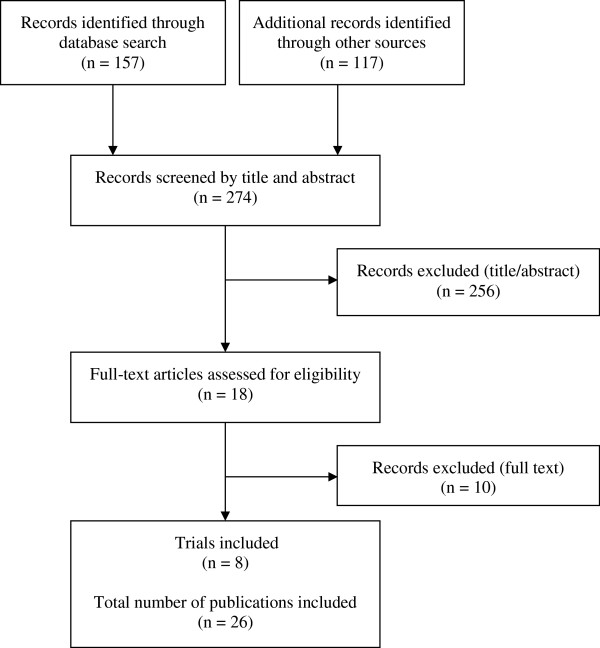
Study selection flow diagram.

A total of 274 publications were identified via database search and forward citation tracking. Eight trials met the inclusion criteria (Figure 1). Main reasons for exclusion were that the interventions were not labelled as complex or the studies did not adhere to the MRC framework. A total of 26 publications were identified as reporting on the eight included trials. In two cases, authors were contacted for missing information. The mean number of publications per trial was 3.25 (range 1 to 6). For one trial, only one publication was available [16]. Four trials were conducted in UK [16-19], two in Ireland [20,21] and one in Germany [22] and the Netherlands [23], respectively. Most of the complex interventions offered structured approaches for improving the quality of care for patients with chronic or complex conditions. Target groups were people suffering from a stroke [16,19], cancer [18,22], coronary heart disease [20], or diabetes mellitus Type 2 [21], patients at risk from prescription and medication management errors in general practice [17], and frail elderly people [23]. The interventions offered improved care concepts or pathways [17,19,20,22] or alternative or modified forms of information or support [16,18,21] e.g. peer support groups for patients and/or their relatives [21]. Table 2 presents an overview of the included trials and the underlying interventions. The quality of reporting on development and piloting of the intervention was judged as good (Table 3). Seven out of eight trials reported on all six items referring to the intervention’s development [17-23] and one trial reported on three items [16]. Most trials presented sufficient information on the feasibility and pilot phase. Concerning the introduction and evaluation phase, the quality of reporting varied. Only one trial reported on all eight items [21] and one trial on seven items [17]. Five trials reported on one to five items [18-20,22,23] and one trial offered no information on any item [16].

**Table 2:** – Overview of included complex interventions

**Reference**	**Aim**	**Country**	**Related publications**
16	Evaluation of a family support organiser (FSO) service for stroke patients and their carers	UK	
17	Evaluation of an information technology intervention for pharmacists to improve prescription safety and medication monitoring in general practices.	UK	Study protocol [24]
			Report for the Department of Health, Patient Safety Research Programme [25]
18	Evaluation of a nurse-delivered intervention for major depressive disorders in patients with cancer	UK	Pilot study [26]
			Intervention development [27]
19	Evaluation of a patient and general practitioner systematic follow-up intervention to improve risk factor management after stroke	UK	Development of the intervention [28]
20	Evaluation of a complex intervention for patients with established coronary heart disease to improve clinical outcomes in general practice	Ireland	Development of the intervention [29]
			Development of the intervention [30]
			Study protocol [31]
			Process Evaluation [7]
			Cost-effectiveness analysis [32]
21	Evaluation of peer support for improving biophysical and psychosocial outcomes for people with type 2 diabetes	Ireland	Development of the intervention [33]
			Study protocol [34]
			Cost-effectiveness analysis [35]
22	Evaluation of an integrated quality of life diagnosis and therapy pathway in breast cancer patients	Germany	Study protocol [36]
			Development of the intervention [37]
			Pilot study [38]
23	Evaluation of a fall prevention program to reduce the fall rate and fear of falling in community-dwelling frail elderly and to alleviate subjective caregiver burden	The Netherlands	Development of the intervention [39]
			Results of the process evaluation [40]

**Table 3:** Results of the rating of the CReDECI items

	[16]	[17]	[18]	[19]	[20]	[21]	[22]	[23]
**Development**								
1. Description of the intervention’s underlying theoretical considerations	+	+	+	+	+	+	+	+
2. Description of all components of the intervention	+	+	+	+	+	+	+	+
3. Rationale for the selection of the intervention’s components	+	+	+	+	+	+	+	+
4. Illustration of any intended interactions between different components	-	+	+	+	+	+	+	+
5. Rationale for the aim/essential functions of the intervention’s components, including the evidence whether the components are appropriate for achieving this goal	-	+	+	+	+	+	+	+
6. Consideration of contextual factors and determinants of the setting in the modelling of the intervention	-	+	+	+	+	+	+	+
**Feasibility and piloting**								
7. Information on pilot-testing	-	+	+	+	+	+	+	+
8. In case of pilot-test: Presentation of all relevant results and their impact on the modelling of the final intervention	n. a.	+	-	+	+	+	+	+
**Introduction of the intervention and evaluation**								
9. Description of the control intervention (comparator)	-	+	-	-	+	+	+	-
10. If the study was conducted in different clusters or centres: Description of a standardised implementation strategy throughout the centres	-	+	n. a.	-	+	+	-	n. a.
11. Description of all materials/ tools used for the implementation of the intervention to allow a replication of the study	-	+	+	+	+	+	+	-
12. Description of an evaluation of the implementation process	-	+	-	-	+^*^	+	-	+
13. Description of any deviation from the study protocol during the implementation process	-	+	-	-	-^*^	+	-	+
14. Description of facilitators or barriers revealed by the process evaluation which have influenced the interventions’ implementation	-	+	-	-	-	+	-	+
15. Description of unexpected interactions between components of the intervention and the environment in which the intervention was implemented	-	-	-	-	-	+	-	-
16. Description of costs or required resources for the intervention’s implementation	-	+	+	-	+	+	-	-^*^

+ Fulfilled; - not fulfilled; n. a. not applicable; * unpublished information delivered by study authors.

The number of items with different ratings per trial ranged from 0–5 with a mean of 2. The two reviewers disagreed on 16 out of the total of 128 ratings (12.5%). The time needed to rate a trial ranged from 30 to 90 minutes, depending on the number of publications per trial.

## Discussion

We identified a small number of trials whose authors explicitly stated having adhered to the MRC framework for developing and evaluating complex interventions [2]. The quality of reporting in these trials was judged as good for the intervention development and piloting phases. Thus, our findings are contrary to previous analyses of the quality of reporting on complex interventions [12,13,41]. However, the trials included in our sample are more likely to fulfil the CReDECI criteria, since all trials confirmed adherence to the MRC framework. For the evaluation phase, quality of reporting varied. While two trials reported on nearly all criteria, six trials offered information only on half of the items or even less. These results confirm the findings from previous studies [9,11,12,42]. Only half of the trials included an evaluation of the implementation process (CReDECI criterion 12). However, this information is required in order to get a deeper understanding of the effects of the evaluation and to describe barriers and facilitators influencing the intervention’s implementation. A process evaluation should be an integral part of the evaluation of complex interventions [2,5,6]. Only half of the trials offered information on the characteristics of the care delivered in the control group, which was often only described as usual or standard care. The lack of information hampers the replication of studies and the adaption of the intervention to different settings or countries [9,10].

The integration of the local context and detailed information on the process evaluation has recently been described as frequently underreported [43,44]. In our sample only a small number of trials included a process evaluation but most trials offered information on the integration of the context in the intervention’s development. However, we did not assess the completeness of the information provided.

The CReDECI list has proven its applicability and practicability. A high rate of agreement was reached by both reviewers. The time needed for assessment varied, depending on the number of publications per trial. Since both reviewers were familiar with complex interventions research and the MRC framework, application of the CReDECI list by untrained users might be more time-consuming.

The currently available reporting guidelines specifically address a defined study design, offering recommendations to improve the reporting of design-specific methodological aspects [45]. The CReDECI list employs a different approach since it comprises items covering three phases of complex interventions’ research: development, feasibility/piloting, and introduction of the intervention and evaluation. However, we recommend the additional use of design-specific reporting guidelines alongside the CReDECI list. Currently, a CONSORT extension for social and psychological interventions is being developed [46]. This extension might be a valuable supplementation to the CReDECI list.

### Limitations of study

Although Medline includes a great number of journals on healthcare research, the search in only one database might be judged as limitation. Further publications not indexed in Medline were identified through forward and backward citation tracking. Thus, it is most likely that we have identified the majority of publications on the development and evaluation of complex intervention explicitly referring to the MRC framework.

In this study, we assessed whether the criteria were fulfilled. Therefore, our analysis offers an overview about relevant aspects of complex interventions’ development and evaluation which were considered in the trials included. However, this analysis offers no judgement on the quality of the information provided.

We included only a small number of trials, since our inclusion criteria were rather strict. Therefore, readers must be aware that the results of our analysis do not represent the reporting quality of publications on complex interventions in general.

## Conclusions

In this study, adhering to the MRC framework for the development and evaluation of complex interventions seems to have a positive impact on the quality of reporting of the complex interventions’ development and feasibility/piloting. These results are in contrast to former analysis [10-13]. Reporting on the evaluation phase could still be improved.

The CReDECI list seems to be a practical instrument for checking the quality of reporting in publications on complex interventions. It could be used alongside established design-specific reporting guidelines such as the CONSORT statement. To further validate the CReDECI list, a formal consensus process with researchers and stakeholders is scheduled.

## Abbreviations

MRC: UK medical research council.

## Competing interests

The authors declared that they have no competing interests.

## Authors’ contribution

Study design: RM, GM; data collection and analysis: RM, GB. Manuscript preparation: RM, GM. All authors read and approved the final manuscript.

## Pre-publication history

The pre-publication history for this paper can be accessed here:

http://www.biomedcentral.com/1471-2288/13/125/prepub

## Supplementary Material

Additional file 1 Complete search strategy. Click here for file
