# Supplementary material for: Quality of reporting of complex healthcare interventions and applicability of the CReDECI list - a survey of publications indexed in PubMed
Source: BMC Med Res Methodol. 2013 Oct 19;13:125. doi: 10.1186/1471-2288-13-125 (PMC3871759; doi:10.1186/1471-2288-13-125)
Supplement: Additional file 1 — Complete search strategy. [file 1471-2288-13-125-S1.pdf]

Complete search strategy

(complex intervention* OR complex nursing intervention*) OR (((MRC OR Medical Research Council) AND framework) OR ((MRC OR Medical Research Council) AND guidance))

Limits: Clinical Trial, Randomized Controlled Trial, English, German, published in the last 10 years
